# Validity of a New Kit Measuring Salivary Lactate Dehydrogenase Level for Screening Gingivitis

**DOI:** 10.1155/2017/9547956

**Published:** 2017-02-26

**Authors:** Daisuke Ekuni, Mayu Yamane-Takeuchi, Kota Kataoka, Aya Yokoi, Ayano Taniguchi-Tabata, Hirofumi Mizuno, Hisataka Miyai, Yoko Uchida, Daiki Fukuhara, Yoshio Sugiura, Takaaki Tomofuji, Manabu Morita

**Affiliations:** ^1^Department of Preventive Dentistry, Okayama University Graduate School of Medicine, Dentistry and Pharmaceutical Sciences, 2-5-1 Shikata-cho, Kita-ku, Okayama, Japan; ^2^Advanced Research Center for Oral and Craniofacial Sciences, Okayama University Dental School, Okayama, Japan

## Abstract

*Aim*. The aim of this study was to determine the usefulness of a new kit that can evaluate salivary lactate dehydrogenase (LD) level in real time for screening gingivitis.* Materials and Methods*. The study included 70 systemic healthy volunteers [29 males and 41 females; mean age ± SD: 24.1 ± 2.6 years]. Resting saliva was collected from each participant and LD level was evaluated in real time using the kit (a color-changing sheet with an integer scale ranging from 1 to 10). A dentist measured probing pocket depth, clinical attachment level, and the proportion of sites with bleeding on probing (% BOP) at six sites on all teeth. Gingivitis was diagnosed when the BOP value was ≥20%.* Results*. Salivary LD level was positively correlated with mean % BOP (odds ratio: 1.47, 95% confidence interval: 1.132–1.916, and *P* < 0.001) in a logistic regression model. The sensitivity and specificity of the kit were 0.89 and 0.98, respectively, at a cut-off value of 8.0 for LD level.* Conclusions*. The new kit for measurement of salivary LD level may be a useful tool to screen for gingivitis in young adults, which contributes to early detection of future periodontitis.

## 1. Introduction

The diagnosis of periodontal disease (gingivitis/periodontitis) has been traditionally based on clinical and radiographic examinations. However, clinical and radiographic examinations are not suitable in large-scale epidemiological studies because they are laborious and costly. The Community Periodontal Index (CPI), developed by the World Health Organization [1], has been widely adopted by public health services. However, its major shortcoming is that the gingival sulcus must be probed by a dentist; this probing may also be painful. Thus, other approaches including salivary diagnostics that can be performed by nonexpert examiners have been suggested [2].

Salivary enzymes, immunoglobulins, and steroid hormones have been proposed as disease markers of periodontal disease [3]. Saliva has been used as an attractive diagnostic fluid of periodontal disease because saliva collection is safe, easy, and noninvasive [4]. Some reviews have also proposed potential salivary markers for periodontal disease [2–5]. Among these disease markers, salivary enzymes are thought to be the most useful biomarkers for the screening and diagnosis of periodontal disease. Candidate enzymes include aspartate aminotransferase, alanine aminotransferase, lactate dehydrogenase (LD), and alkaline phosphatase [6].

LD is an enzyme that is detectable in the cytoplasm in almost every cell of the human body and its extracellular presence is always related to tissue breakdown [7]. Major salivary glands contribute very little to LD level in whole saliva [8]. Therefore, the main source of LD in whole saliva is thought to be the oral epithelium [7], which suggests that salivary LD level may be a feasible and useful parameter for screening periodontal disease [6, 7, 9–11]. However, salivary LD levels are variable because of the sampling, handling, and analytical methods used [7]. Furthermore, specialized laboratory equipment is required for measurement. Taken together, to utilize salivary LD as a screening tool, a quick and simple measurement system is needed.

The mildest form of periodontal disease, that is, gingivitis, has a simple pathology. The inflammatory status is caused by an oral bacterial biofilm (dental plaque) that accumulates on teeth adjacent to the gingiva. If gingivitis is not appropriately treated, it leads to periodontitis, which then induces alveolar bone and tooth loss. Since gingivitis is reversible, early detection of gingivitis plays a significant role in successful clinical treatment and ultimately leads to a greater tooth survival rate. Controlling gingivitis as early as possible may reduce any severe impacts later on in life. Thus, we focused on screening of gingivitis in this study.

Recently, a new and simple test kit to assess salivary LD level within 1 minute has been developed and is commercially available. It can detect LD level without using specialized laboratory devices. However, no studies have been conducted on the usefulness of this new test kit for screening gingivitis. The aim of the present study was to investigate the association between salivary LD level and gingivitis and determine the feasibility and reliability of this new kit for screening gingivitis in young adults.

## 2. Materials and Methods

### 2.1. Participants

A total of 71 university students without any systemic diseases and not receiving any medication voluntarily participated in the present study. We excluded one current smoker because smoking habit has been shown to affect LD level [8]. Finally, 70 systemic healthy participants (29 males and 41 females; mean age ± SD, 24.1 ± 2.6 years; range, 20–33 years) were analyzed. The recruitment period was from December 2014 to April 2015 and from October 2015 to November 2015. The study was approved by the Ethics Committee of Okayama University Graduate School of Medicine, Dentistry and Pharmaceutical Sciences (numbers 808 and 1060). Written informed consent was obtained from all participants.

### 2.2. Measurement of Salivary LD Level

**Figure 1:**
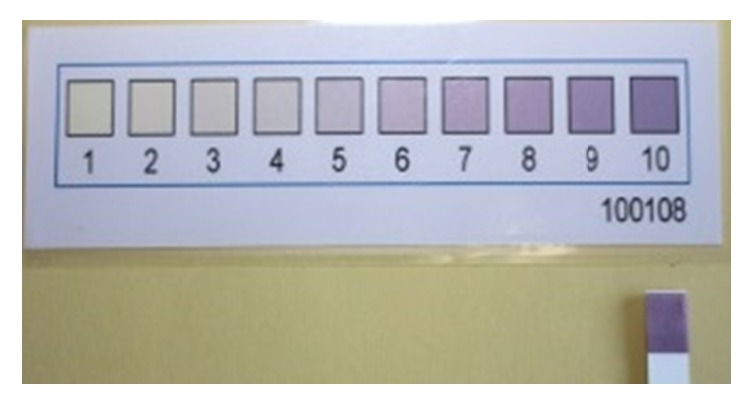
The new kit for measurement of salivary LD level. The kit has a color-changing sheet that uses an integer scale ranging from 1 to 10 according to the LD level. The scale guide and a representative sample are shown.

The salivary LD level was measured using a commercially available kit (PD-1, Nagata Corp., Shiso, Japan). The kit consists of a reagent strip that includes 3.347 mg/mL nicotinamide adenine dinucleotide, 500 U/mL diaphorase, 5.0 mg/mL nitroblue tetrazolium, 12 mg/mL Tris buffer, 40 mg/mL Li lactate, and 10 mg/mL bovine serum albumin. In the presence of LD, formazan (purple color) is produced from nitroblue tetrazolium (faint yellow). Briefly, approximately 0.5 mL of resting whole saliva was collected from each participant and immediately applied to the reagent strip according to the manufacturer's protocol. The color change, which indicates LD level, was recorded after 1 minute according to the kit's scale guide (Figure 1). Two trained dentists (Mayu Yamane-Takeuchi and Aya Yokoi) evaluated the color change simultaneously and determined the color value together. The kappa coefficients for intra- and interexaminer reliability were >0.8. To assess the reproducibility of the kit, 1–1,600 U/L LD (Cayman Chemical, Ann Arbor, MI, USA) was used for calibration. Measurement was performed in triplicate. Both intra- and interassay coefficients of variation were <5%.

To investigate the influence of tooth brushing, a preliminary study was performed in which whole resting saliva was collected from eight participants at baseline and 5 minutes after tooth brushing and mouth rinsing. The change in color was within ±1 value. In addition, diurnal variations [morning (between 8 : 00 and 9 : 00), noon (between 12:00 and 1 : 00), and evening (between 16:00 and 17:00)] were examined in another six participants. The change in color was within ±1 value.

### 2.3. Oral Examination

After the measurement of LD level, probing pocket depth (PPD) and clinical attachment level (CAL) were determined at six sites (mesiobuccal, midbuccal, distobuccal, mesiolingual, midlingual, and distolingual) on all teeth using a color-coded probe (CP-11 Color-Coded Probe, Hu-Friedy, Chicago, IL, USA) [12]. The proportion of sites with bleeding on probing (% BOP) was also measured in each participant. The Plaque Control Record (PCR) was measured using erythrosine staining and recorded with respect to the relative location to the gingival margin at four sites (mesial, distal, buccal, and lingual) around each tooth [13]. The presence of an inflammatory lesion or injury in the oral cavity, which may affect LD level, was also assessed by inspection and palpation. All clinical procedures were performed by a trained dentist (Daisuke Ekuni).

### 2.4. Questionnaire

The questionnaire included the following items: age, sex, medication, general condition, smoking, and timing of tooth brushing before the oral examination.

### 2.5. Sample Size Estimation

Power analysis and sample size were calculated using statistical software (SamplePower ver. 3.0, IBM, Tokyo, Japan) based on the results of salivary LD level from the preliminary study. A two-way table was calculated to detect positive or negative differences in salivary LD level (Appendix). The minimum sample size in both the positive and negative groups to detect statistically significant differences in salivary LD level was 9 with 80% power and a two-sided 5% significance level. Therefore, enrollment was stopped after 9 participants with gingivitis were enrolled.

### 2.6. Statistical Analysis

Data analysis was performed using the Statistical Package for Social Science (SPSS ver. 23, SPSS Japan, Tokyo, Japan). Levels of significance were set at *P* < 0.05. Gingivitis was defined when % BOP was ≥20% of sites (six sites per tooth) without PPD ≥5 mm and CAL ≥2 mm at all sites [14]. Healthy gingival tissue as control was defined when % BOP was <20% of sites without PPD ≥5 mm and CAL ≥2 mm [14]. To determine the cut-off points for LD level, receiver operating characteristic curves (ROC curves) were constructed and points showing minimum differences between sensitivity and specificity were determined for gingivitis. Fisher's exact test was performed to test for significant differences, and positive and negative predictive values were calculated for LD level. Fisher's exact test, Mann–Whitney* U* test, or an unpaired* t*-test was performed to compare parameters observed in healthy and gingivitis groups. Associations between the LD level and other parameters were analyzed using Spearman's rank correlation' coefficients. A backward, stepwise multinomial logistic regression analysis for all participants was also performed to evaluate relationships between LD level and other variables. To control for confounding variables, the final model included variables with *P* < 0.2 in bivariate analyses [15]. The LD level ≥ 8.0 or not was used as the dependent variable, while age, number of natural teeth present, PPD, BOP, and PCR were used as the independent variables in the analysis. The odds ratio (OR) and 95% confidence interval (CI) were calculated. The logistic regression models were reviewed for goodness-of-fit and validated using the Hosmer-Lemeshow statistic [16, 17].

## 3. Results

Table 1 shows the participant characteristics. No participants had any inflammatory lesion or obvious injury in the oral cavity, except for gingivitis, and had periodontitis. Significant differences in the percentage of males, salivary LD level, mean PPD, BOP, and PCR between the healthy and gingivitis groups were observed (*P* < 0.05 for all) (Table 2).

**Figure 2:**
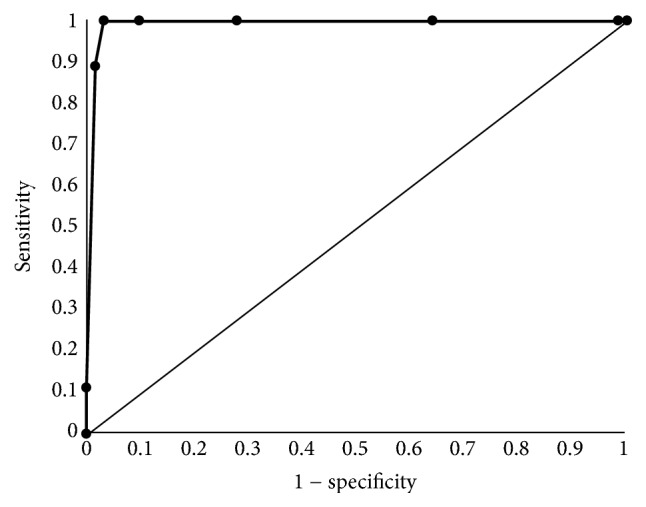
Receiver operating characteristic curves for detection of gingivitis.

To evaluate the discrimination power of salivary LD level, ROC curves were constructed for screening gingivitis (Figure 2). When the cut-off value for LD level was set at 8.0, as determined by ROC analysis, the area under the curve was 0.991 (95% confidence interval: 0.972–1.000) (*P* < 0.001). The sensitivity, specificity, and positive and negative predictive values were 0.89, 0.98, 0.89, and 0.98, respectively. Under this condition, 88.9% of participants with gingivitis were detected (Table 3).

Spearman's rank correlation' coefficients between the LD level and other parameters are shown in Table 4. The LD level was significantly associated with number of natural teeth present, PPD, % BOP, and PCR (*P* < 0.05 for all) (Table 4). In the logistic regression analysis, the LD level was positively correlated with % BOP (*P* < 0.001) (Table 5).

## 4. Discussion

In this study, evaluation of salivary LD level using a new kit showed high diagnostic performance and acceptable scores for screening subjects with gingivitis (sensitivity: 0.89 and specificity: 0.98) among young adults. Accordingly, 88.9% of participants with gingivitis were detected using an LD level cut-off value of 8.0. Our results support previous studies that described salivary LD level which may be a feasible and useful parameter for screening periodontal disease [6, 7, 9–11].

Periodontal disease is one of the most prevalent oral diseases among middle-aged and elderly populations. Since gingivitis is reversible and can be managed by appropriate plaque control [18], early detection of gingivitis can prevent the progression of periodontal disease (gingivitis to periodontitis). Gingivitis is a “silent disease” and many people, including university students, cannot accurately detect their deteriorating periodontal condition [19]. Our findings suggest that the new kit is a useful tool to screen for gingivitis, which can help to prevent future periodontitis.

For large-scale epidemiological surveys, cost-effective screening methods are required. In Japan, the CPI has been commonly applied in mass checkups [11]. However, the CPI has some limitations. First, the CPI requires probing, which induces pain and puts patients with periodontitis at risk of bacteremia [20]. Second, the examination must be completed by well-trained dentists, which adds to the cost of evaluation [11]. Thus, the CPI is not cost-effective for mass screening. Conversely, with the new test results are ready within 1 minute and it only costs 200 Japanese yen, which is less than 2 US dollars. The new test kit has a low time and cost burden on participants. Furthermore, well-trained dentists and specialized laboratory equipment are not necessary, which is advantageous for mass screening.

In Japan, health examinations are performed on a routine basis according to the School Health and Safety Act. However, oral health examinations are not mandatory for university students. The Industrial Safety and Health Act stipulates that Japanese companies must offer annual health examinations for all employees. However, the oral health examination is optional. In fact, many companies do not offer oral health examinations because they are costly and time-consuming [11]. Our screening method may resolve these problems in both school and occupational fields. In addition, salivary LD level can be a predictive marker of healthcare costs [21], which may further help to cut costs.

In this study, we focused on gingivitis but not periodontitis. However, a recent study on salivary LD [11] indicated a relatively lower sensitivity (0.709) and specificity (0.711) for screening periodontitis. This finding may be because of the multifactorial characteristic of periodontitis. Chronic periodontitis has divergent biological phases and the expression of individual salivary biomarkers fluctuates during the course of the disease [22]. As a recent review suggests that “the bacterial burden, inflammatory response, and tissue destruction may not occur simultaneously at one site of the periodontal tissue or in the whole mouth” [2], salivary LD level may be associated with a specific biological stage of periodontitis, such as increased soft tissue destruction, but not with more severe stages, such as alveolar bone loss. Furthermore, the concentrations of some biomarkers may be altered in the presence of local and systemic factors, such as smoking, cardiovascular disease or diabetes [2, 23–25]. Therefore, LD measurement might be more useful in screening gingivitis in younger populations with relatively fewer confounding factors.

The association between other salivary markers and gingivitis was investigated in previous studies [26–32]. Among them, only one study reported the sensitivity (0.76) and specificity (0.45) of salivary leukocytes for screening gingivitis [28]. These values were lower than our results, suggesting that detection of salivary LD level may be more suitable for screening gingivitis in young adults.

In the logistic regression analysis, only % BOP was positively correlated with salivary LD level. BOP is a widely used indicator to show the presence of inflammation [33, 34] and an earlier and more sensitive indicator of inflammation than PPD or visual signs of inflammation (redness and swelling) [35]. The absence of BOP is considered a good predictor of periodontal stability [36]. Whereas BOP may be a poor indicator of disease progression [34, 36, 37], a meta-analysis suggested that a repeatedly BOP-positive site is associated with a significantly increased risk for attachment loss or periodontal disease progression [38]. Based on these findings, if salivary LD activities are repeatedly high in young adults, then progression of periodontal disease may occur in the future.

In this study, nine participants (12.9%) had gingivitis. We defined gingivitis using three parameters (% BOP, PPD, and CAL) according to a previous study [15]. Based on a national survey of dental diseases conducted by the Japanese Ministry of Health, Labour, and Welfare in 2005, 9.8–14.0% of participants aged 20–34 years had a BOP-positive tooth corresponding to CPI Code 1. Although there are differences in the methods and participant characteristics between our study and the national survey, the prevalence rate in our study was within the expected range. Thus, our findings may be generalizable to young Japanese populations. However, as all participants were recruited at Okayama University, sampling bias should be taken into consideration.

There are some limitations associated with this study. First, this was a cross-sectional study. As mentioned above, as repeatedly high LD level may be an indicator of disease progression, a prospective cohort or intervention study is required. Second, the number of participants was small and we only analyzed systemic healthy participants and nonsmokers. Therefore, large-scale studies are necessary to confirm our results.

## 5. Conclusion

The new kit, which measures salivary LD level, may be a useful tool to screen for gingivitis, which contributes to early detection of future periodontitis.

## Figures and Tables

**Table 1:** Characteristics of participants (*n* = 70).

Variable	Number (%) or mean ± SD
% male	29 (41.4)
Age (year)	24.1 ± 2.6
Salivary lactate dehydrogenase level	4.6 ± 1.7
Number of natural teeth present	28.2 ± 1.9
Mean probing pocket depth (mm)	1.9 ± 0.2
Bleeding on probing (%)	10.9 ± 8.8
Plaque Control Record (%)	51.7 ± 23.9

**Table 2:** Differences between healthy and gingivitis groups (*n* = 70).

Variable	Healthy (*n* = 61)	Gingivitis (*n* = 9)	*P* value^§^
% male	22 (36.1)^*∗*^	7 (77.8)	0.018
Age (year)	24.0 ± 2.7^†^	24.4 ± 2.4	0.651
Salivary lactate dehydrogenase level	4.0 (3.0, 5.0)^‡^	8.0 (8.0, 8.0)	<0.001
Number of natural teeth present	28.0 ± 1.9	29.2 ± 1.9	0.088
Mean probing pocket depth (mm)	1.8 ± 0.1	2.1 ± 0.2	<0.001
Bleeding on probing (%)	7.7 ± 4.0	30.9 ± 4.3	<0.001
Plaque Control Record (%)	49.8 ± 24.3	64.4 ± 16.8	0.087

^*∗*^Number (%).

^†^Mean ± standard deviation.

^‡^Median (25%, 75%).

^§^Fisher's exact test, Mann–Whitney *U* test, or unpaired *t*-test.

**Table 3:** Definition of gingivitis group compared with healthy group.

Salivary lactate dehydrogenase level	Healthy (*n* = 61)	Gingivitis (*n* = 9)	Total
<8.0	60	1	61
≥8.0	1	8	9

*P* < 0.001; Fisher's exact test.

**Table 4:** Spearman's rank correlation' coefficients between LD level and independent variables (*n* = 70).

Independent variables	*ρ* ^*∗*^ between LD and independent variables	*P* value
Age (year)	0.175	0.175
Number of natural teeth present	0.261	0.029
Mean probing pocket depth (mm)	0.483	<0.001
Bleeding on probing (%)	0.777	<0.001
Plaque Control Record (%)	0.288	0.015

^*∗*^Spearman's rank correlation' coefficients.

LD: lactate dehydrogenase.

**Table 5:** A stepwise, backward multinomial logistic regression analysis with lactate dehydrogenase level as the dependent variable (*n* = 70).

Variables	Adjusted OR^*∗*^	95% CI	*P* value
Bleeding on probing (%)	1.473	1.132–1.916	0.004

OR: odds ratio; CI: confidence interval; ^*∗*^adjusted for number of natural teeth present, mean probing pocket depth, bleeding on probing, and Plaque Control Record.

**Table 6:** A result in the preliminary study.

Salivary lactate dehydrogenase level	Healthy	Gingivitis	Total
	(*n* = 15)	(*n* = 3)	
Negative	14	1	15
Positive	1	2	3

## Appendix

See Table 6.
